# Disclosing the Potential of the SARP-Type Regulator PapR2 for the Activation of Antibiotic Gene Clusters in Streptomycetes

**DOI:** 10.3389/fmicb.2020.00225

**Published:** 2020-02-18

**Authors:** Janina Krause, Ira Handayani, Kai Blin, Andreas Kulik, Yvonne Mast

**Affiliations:** ^1^Department of Microbiology/Biotechnology, Interfaculty Institute of Microbiology and Infection Medicine, Faculty of Science, University of Tübingen, Tübingen, Germany; ^2^Research Center for Biotechnology, Indonesian Institute of Sciences (LIPI), Cibinong, Indonesia; ^3^Novo Nordisk Foundation Center for Biosustainability, Technical University of Denmark, Lyngby, Denmark; ^4^German Center for Infection Research (DZIF), Partner Site Tübingen, Tübingen, Germany; ^5^Department of Bioresources for Bioeconomy and Health Research, Leibniz Institute DSMZ – German Collection of Microorganisms and Cell Cultures, Braunschweig, Germany; ^6^Department of Microbiology, Technical University Braunschweig, Braunschweig, Germany

**Keywords:** actinomycetes, *Streptomyces*, antibiotic, regulator, SARP, silent gene cluster

## Abstract

*Streptomyces* antibiotic regulatory protein (SARP) family regulators are well-known activators of antibiotic biosynthesis in streptomycetes. The respective genes occur in various types of antibiotic gene clusters encoding, e.g., for polyketides, ribosomally and non-ribosomally synthesized peptides, or β-lactam antibiotics. We found that overexpression of the SARP-type regulator gene *papR2* from *Streptomyces pristinaespiralis* in *Streptomyces lividans* leads to the activation of the silent undecylprodigiosin (Red) gene cluster. The activation happens upon the inducing function of PapR2, which takes over the regulatory role of RedD, the latter of which is the intrinsic SARP regulator of Red biosynthesis in *S. lividans*. Due to the broad abundance of SARP genes in different antibiotic gene clusters of various actinomycetes and the uniform activating principle of the encoded regulators, we suggest that this type of regulator is especially well suited to be used as an initiator of antibiotic biosynthesis in actinomycetes. Here, we report on a SARP-guided strategy to activate antibiotic gene clusters. As a proof of principle, we present the PapR2-driven activation of the amicetin/plicacetin gene cluster in the novel Indonesian strain isolate *Streptomyces* sp. SHP22-7.

## Introduction

In 2018, the WHO warned that the dramatic increase of antibiotic resistances coupled with the scarcity of new antibiotics will lead to a global health crisis in the 21st century (World Health Organization, 2015). Even nowadays, infections that are caused by drug-resistant pathogens are suggested to account for 700,000 deaths worldwide annually (United Nations Foundation and the Wellcome Charitable Trust, 2016). According to the World Bank Group “by 2050, drug-resistant infections could cause global economic damage on par with the 2008 financial crisis” (Worldbank, 2016). Thus, there is a substantial need for new antibiotics in order to combat drug-resistances. Bacteria have long been recognized as a prolific source for antibiotics (Newman and Cragg, 2016). Especially actinomycetes are potent producers of bioactive molecules, as they provide up to 70% of all medically important antibiotic agents (Tanaka and Omura, 1990). The capability to produce these natural compounds is genetically encoded in the actinomycetes genome, whereby the respective genes usually are organized as biosynthetic gene clusters (BGCs). In recent years, genomic analyses of actinomycetes have revealed the presence of numerous “silent” or “cryptic” BGCs, meaning that these clusters remain silent or are only weakly expressed under standard lab conditions. Indeed it is estimated that actinomycetes encode ∼10-times the number of secondary metabolites than anticipated from prior fermentation studies (Baltz, 2017). Thus, these microorganisms still hold the genetic potential to produce new bioactive compounds. Consequently, there are several attempts to activate silent gene cluster expression in order to find new antibiotics. However, most of these activation efforts are either (a) completely unspecific in terms of the BGC(s) to be activated [e.g., by adding general elicitors to the cell culture, co-cultivation approaches, or strain-cultivations following the “one strain-many compounds” (OSMAC) strategy] or (b) they are absolutely specific for the BGC of interest (e.g., heterologous expression of the BGC, introduction of an artificial promoter in front of the BGC, or manipulation of a cluster-situated regulator) as reviewed in Ochi and Hosaka (2013) and Zhu et al. (2014). Both approaches have their drawbacks, as there are either major analytical efforts to identify the product from the silent gene cluster [in terms of (a)] or tedious genetic engineering efforts to manipulate the producer strain [in terms of (b)]. Thus, it would be highly beneficial to have a more general activation strategy that targets a defined set of BGCs, which would tackle both issues. In a recent study from Martínez-Burgo et al. (2019) it has been shown that conserved pathway-specific activators can be used to activate BGC expression in a foreign *Streptomyces* strain. In this study the heterologous expression of the PAS-LuxR type regulator gene *pimM* from *Streptomyces natalensis* in *Streptomyces clavuligerus* led to the activation of clavulanic acid, cephamycin C, and tunicamycin production.

Here we demonstrate that *Streptomyces* Antibiotic Regulatory Protein (SARP)-type regulators can be used as activators of certain antibiotic gene clusters in actinomycetes and describe a genome-based approach to screen for SARP-activated gene clusters. SARPs have exclusively been found in actinomycetes, especially in streptomycetes, where they act as pathway-specific activators of secondary metabolite biosynthesis (Bibb, 2005). They are known to be associated with various antibiotic gene clusters, encoding type I- (Bate et al., 2002; Takano et al., 2005; Novakova et al., 2011) and type II-PKS derived polyketides (Lombó et al., 1999; Sheldon et al., 2002; Aigle et al., 2005; Novakova et al., 2011), ribosomally (Widdick et al., 2003; Wu et al., 2018) and non-ribosomally synthesized peptides (Ryding et al., 2002), hybrid polyketide-peptide compounds (Pulsawat et al., 2009; Suzuki et al., 2010; Xie et al., 2012; Salehi-Najafabadi et al., 2014; Mast et al., 2015; Ye et al., 2018), β-azachinones (Santamarta et al., 2002; Rodríguez et al., 2008; Kurniawan et al., 2014), and azoxy compounds (Garg et al., 2002). SARP genes usually are located within the BGC they are regulating. The encoded SARP gene products are characterized by a winged helix-turn-helix (HTH) DNA-binding motif at the N-terminus that binds to a conserved recognition sequence within the major groove of the target DNA (Wietzorrek and Bibb, 1997; Liu et al., 2013). The DNA recognition sequence constitutes direct heptameric repeat sequences followed by 4 bp spacers, which are often localized between the −10 and the −35 promoter element of the respective target DNA. Such a localization has already been described for the SARP type regulator AfsR from *Streptomyces coelicolor*, which binds to a recognition sequence 8 bp upstream of the −10 element (Tanaka et al., 2007). Also the SARP regulators Aur1PR4 from *Streptomyces aureofaciens* and FdmR1 from *Streptomyces griseus* bind to heptameric repeat sequences, which are located 8 bp upstream of the −10 region (Chen et al., 2008; Rehakova et al., 2013). ActII-ORF4 from *S. coelicolor* interacts with the −35 element for transcriptional activation (Arias et al., 1999). DnrI from *Streptomyces peucetius* and SanG from *Streptomyces ansochromogenes* bind to interaction sites that occur within the −35 element (Sheldon et al., 2002; He et al., 2010). It is suggested that in general the SARP binding site overlaps with the −35 region of the target promoter, which is a binding region of the majority of repressors but not activators. Thus, SARP-driven transcriptional activation has been proposed to occur via a novel mechanism (Tanaka et al., 2007). The C-terminal bacterial activation domain (BTAD) of the SARP protein activates the transcription of the target genes by recruiting the RNA polymerase (RNAP) to the respective promoter, where a ternary DNA–SARP–RNAP complex is formed allowing for transcriptional initiation (Tanaka et al., 2007). “Small” SARP-type activators only contain the HTH DNA binding and BTAD domain, whereas “large” SARPS carry additional domains at the C-terminal side of the protein. These domains include a domain of unknown function belonging to the P-loop NTPase family, and one or more copies of a tetratricopeptide repeat (TPR) motif (Liu et al., 2013). A typical “small” SARP-type activator is represented by PapR2, which has been identified as the major activator of pristinamycin biosynthesis in *Streptomyces pristinaespiralis* (Mast et al., 2015). A *papR2* deletion mutant is unable to produce any pristinamycin, depicting that PapR2 is essential for pristinamycin biosynthesis (Mast et al., 2015). In contrast, overexpression of *papR2* in *S. pristinaespiralis* leads to an increased pristinamycin production, which shows that PapR2 has an activating function (Mast et al., 2015). With the help of electromobility shift assays (EMSAs) and (quantitative) reverse transcription PCR [RT-(q)PCR] analysis the PapR2 target genes have been identified in the pristinamycin producer and a conserved PapR2 binding site was proposed (Mast et al., 2015).

In this study, we report on the potential of SARP-type regulators as genetic engineering devices for the activation of (silent) BGCs in actinomycetes. SARP-type regulators are present predominantly in actinomycetes with an abundance of 98% in the genus *Streptomyces.* We demonstrate that the SARP-type regulator PapR2 activates the silent undecylprodigiosin (Red) gene cluster in *Streptomyces lividans.* Additionally, we provide evidence for a PapR2-guided activation of a BGC in the poorly studied Indonesian strain isolate *Streptomyces* sp. SHP22-7 (SHP22-7), which yielded an increased production of the nucleoside antibiotic plicacetin.

## Materials and Methods

### Bacterial Strains, Plasmids, and Cultivation Conditions

The bacterial strains and plasmids used in this study are listed in Supplementary Table S1. For routine cloning strategies *Escherichia coli* Novablue (Novagen) was used. *S. lividans* T7 (Fischer, 1996) and SHP22-7 (Handayani et al., 2018) were applied for antibiotic production analysis, generation of overexpression strains, and transcriptional analysis. Cloning procedures and strain cultivation were carried out as described before (Mast et al., 2015). For cultivation and isolation of RNA, *Streptomyces* strains were grown in 100 ml of R5 medium in 500-ml Erlenmeyer flasks (with steel springs) on an orbital shaker (180 rpm) at 28°C (Kieser et al., 2000). For isolation of genomic DNA and protoplast transformation experiments, strains were grown in 100 ml of S-medium (Kieser et al., 2000). Kanamycin (50 μg/ml), apramycin (50 μg/ml), or thiostrepton (20 μg/ml) were used for selection when appropriate. For antibiotic production experiments with *S. lividans*, strains were grown in YEME medium as reported before (Mast et al., 2015). For antibiotic production experiments with SHP22-7, strains (SHP22-7*papR2-OE*; references: SHP22-7*pRM4* and SHP22-7 WT) were grown in 50 ml NL410 medium consisting of glucose (10 g l^–1^), glycerol (10 g l^–1^), oat meal (5 g l^–1^), soy flour (10 g l^–1^), yeast extract (5 g l^–1^), Bacto casamino acids (5 g l^–1^), CaCO_3_ (1 g l^–1^), and distilled water (pH was adjusted to 7.0 with NaOH) as a preculture. After 3 days, 10 ml of preculture was transferred to 100 ml of sterile main culture medium NL19, consisting of mannitol (20 g l^–1^), soy flour (20 g l^–1^), and distilled water (pH adjusted to pH 7.5 with NaOH). Cells were grown for 168 h at 28°C.

### Molecular Cloning

Basic procedures for DNA manipulation were performed as described previously (Sambrook et al., 1989; Kieser et al., 2000). Primers used for PCR were obtained from MWG Biotech AG (MWG, Ebersberg, Germany) and are listed in Supplementary Table S1.

### Construction of the papR2 Overexpression Strain SHP22-7papR2-OE

For *papR2* overexpression experiments with SHP22-7, the *papR2* gene was isolated as a *Nde*I/*Hin*dIII-fragment from plasmid pGM190/papR2 (Mast et al., 2015) and was cloned into the *Nde*I/*Hin*dIII restriction site of the integrative expression vector pRM4. In the resulting overexpression construct pRM4/papR2, the *papR2* gene is under control of the constitutive promoter of the erythromycin resistance gene *ermEp*^∗^. pRM4/papR2 was transferred to SHP22-7 by protoplast transformation. Transformants were selected with apramycin (50 μg/ml), which resulted in the overexpression strain SHP22-7*papR2-OE.* Strain SHP22-7*pRM4*, harboring the empty pRM4 vector, was generated by protoplast transformation accordingly and served as a reference.

### PapR2 Protein Expression in *S. lividans*

For *papR2* overexpression experiments *SLpapR2-OE* precultures were grown in 100 ml of YEME liquid medium for 2 days at 28°C. Five milliliters of preculture was used as inoculum for 100 ml YEME liquid medium as main culture with thiostrepton (12.5 μg/ml) as inductor for gene expression (Mast et al., 2015). The main culture was cultivated for 3 days at 28°C. PapR2 protein purification was carried out as reported before (Mast et al., 2015).

### Spectrophotometrical Analysis for Red Detection

For Red detection, culture supernatant from *SLpapR2-OE* and *SLpGM190* (reference) was treated as reported in Onaka et al. (2011) and absorption was measured with a Hitachi U-2000 spectrophotometer.

### Sample Treatment for SHP22-7 Bioassays and Compound Detection

For SHP22-7 compound detection and bioassay tests, 5 ml culture samples of SHP22-7*papR2-OE* (references: SHP22-7*pRM4* and SHP22-7 WT) was extracted with 5 ml ethyl acetate for 30 min at RT. Ethyl acetate samples were concentrated *in vacuo* completely and then redissolved in 0.75 ml of methanol. Methanolic extracts were used for bioassays and high-performance liquid chromatography/mass spectrometry (HPLC–MS) analysis.

### Bioassays

Antibiotic activity was analyzed in disc diffusion assays using *Bacillus subtilis* ATCC6051 as test organism. Thirty microliters of methanolic extract from three independent biological samples of SHP22-7 WT, SHP22-7*pRM4*, and SHP22-7*papR2-OE*, respectively, was pipetted on a filter disc, which was placed on a *B. subtilis* test plate. Five microliters of kanamycin (50 μg/ml) was applied as a positive control and 30 μl of methanol as negative control to test the functionality of the *B. subtilis* bioassay plates. The plates were incubated overnight at 37°C. Antibiotic activity was quantified by measuring the diameter of the inhibition zone around the filter discs. The bioassay was carried out as 10 independent biological replicates.

### HPLC and HPLC–MS Analysis for Amicetin/Plicacetin Detection

High-performance liquid chromatography analyses were performed with a HP1090M system with ChemStation 3D software rev. A.08.03 (Agilent Technologies, Waldbronn, Germany) on a Nucleosil C18 column (5 μm, 125 mm × 3 mm) fitted with a precolumn (20 × 3 mm) and with a flow rate of 850 μl min^–1^. Chromatography was done by linear gradient elution from 95.5 solvent A (water with 0.1% phosphoric acid) to 100% solvent B [acetonitrile (ACN)] over 15 min. The injection volume was 5 μl. Multiple wavelength monitoring was performed at 210, 230, 260, 280, 310, 435, and 500 nm. UV-Vis spectra were measured from 200 to 600 nm. The evaluation of the chromatograms (210 nm only) was done by means of an in-house HPLC–UV–Vis database.

High-performance liquid chromatography–mass spectrometry analysis of amicetin/plicacetin was performed with an Agilent 1200 series chromatography system (binary pump, high performance autosampler, DAD-detector) coupled with an LC/MSD Ultra Trap System XCT 6330 (Agilent Technologies, Waldbronn, Germany). The sample (5 μl) was injected on a Nucleosil 100 C18 column (3 μm, 100 × 2 mm) fitted with a precolumn (3 μm, 10 × 2 mm) at a flow rate of 400 μl/min and a linear gradient from 100% solvent A (0.1% HCOOH in water) to 100% solvent B (0.06% HCOOH in ACN) over 15 min at 40°C. UV–Vis-detection was done at 220, 260, 280, 360, and 435 nm. Electrospray ionization was performed in positive and negative ultra-scan mode (alternating) with a capillary voltage of 3.5 kV and a drying gas temperature of 350°C. Detection of *m/z* values was conducted with Agilent DataAnalysis for 6300 Series IonTrap LC/MS Version 3.4 (Bruker Daltonik). Upon HPLC–MS analyses amicetin, plicacetin, and plicacetin isomer were identified by comparisons of their UV/visible spectra, retention times, and molecular masses with authentic standards, as *m/z* 617.1 [M−H]^–^ and *m/z* 516.1 [M−H]^–^, respectively.

### AntiSMASH Analysis

With the webtool antiSMASH whole genomes can be scanned for the occurrence of BGCs. Gene cluster similarity is given in % and indicates the number of similar genes to a known cluster. Genes are similar if a BLAST-alignment results in an *e*-value <10^–5^ and the sequence identity is >30%. Additionally, the shortest alignment must enclose >25% of the sequence. If all genes of a known cluster can be found in the query cluster, the similarity of the sequences is 100%. The similarity lowers if less genes of the known cluster can be found in the query cluster (Medema et al., 2011).

### PatScan Analysis

PatScan analysis (Blin et al., 2018) was performed with the *S. lividans* T7 genome (GenBank Accession Number ACEY00000000) and the PapR2 consensus sequence 5′-GTCAGSS-3′ using the software at https://patscan.secondarymetabolites.org/.

### PapR2 Electromobility Shift Assays

For EMSAs with Red-specific promoter regions, 182 bp DNA fragments of the upstream regions of *redP* and *redQ* were amplified by PCR from genomic DNA of *S. lividans* T7 with primer pairs PredPfw/rv and PredQfw/rv, respectively (Supplementary Table S1). For EMSAs with the *pliA* promoter region, a 230 bp DNA fragment of the upstream region of *pliA* was amplified by PCR from genomic DNA of strain SHP22-7 as template and primer pair PpliAfw/rv (Supplementary Table S1). Promoter DNA amplificates included a 16 bp Cy5 adapter sequence, each at the 3′- and 5′-end, which was added via the respective primer sequences. The generated amplificates were used as templates in a second PCR approach together with a Cy5 primer (Supplementary Table S1) in order to conduct Cy5 labeling of the promoter regions. Promoter labeling and PapR2 EMSAs were carried out with variable concentrations of PapR2 protein sample as reported before (Mast et al., 2015). To verify the specificity of the PapR2-DNA binding, an excess of unlabeled, specific, and non-specific DNA, respectively, was added to the EMSA mixture as described previously (Mast et al., 2015). DNA bands were visualized by fluorescence imaging using a Typhoon Trio^TM^ Variable Mode Imager (GE Healthcare).

### Transcriptional Analysis by Reverse Transcription Analysis (RT-PCR)

*SLpGM190* and *SLpapR2-OE*, as well as SHP22-7, SHP22-7*pRM4*, and SHP22-7*papR2-OE* were each grown under *papR2*-overexpression conditions as described above. Thirty milliliters of each cell culture was harvested after 48 h. Cell disruption was carried out with glass beads (150–212 μm; Sigma) at 6,500 rpm, 1 × 20-30 s, using a Precellys Homogenizer (Peqlab). Total RNA was isolated as described previously (Sambrook et al., 1989) and served as the basis for RT-PCR experiments. DNA was removed by digestion with DNase (Thermo Fisher Scientific) and absence of DNA was verified via PCR analysis. RNA concentrations and quality were checked using a NanoDrop ND-1000 spectrophotometer (Thermo Fisher Scientific). cDNA from 3 μg RNA was generated with random primers, reverse transcriptase, and cofactors (Fermentas). For RT-PCRs, primers were used that amplify cDNA of 200–250 bp from internal gene sequences. PCR conditions were 98°C for 5 min, followed by 30 cycles of 95°C for 30 s, 60°C for 30 s, and 72°C for 45 s, and a final cycling step at 72°C for 5 min. As a positive control, cDNA was amplified from the 16S rRNA transcript, which is transcribed constitutively. To exclude DNA contamination, negative controls were carried out by using total RNA as a template for each RT-PCR reaction. At least three independent biological replicates have been tested.

### Transcriptional Analysis by Real-Time qPCR

Real-time qPCR analysis was applied for quantitative cDNA determination. PCR reactions were run with SYBR^®^ Green Supermix (BioRad) on an iQ5 Multicolor Real-Time-PCR Detection System (BioRad). The SYBR^®^ Green Dye shows increased fluorescence when bound to double-stranded DNA. The fluorescence is measured at 494 and 521 nm and gives the proportional amount of generated dsDNA. cDNA was generated from cultures of *SLpGM190* and *SLpapR2-OE* as described above. The primer pairs redPintfw/rv and redQintfw/rv, respectively (Supplementary Table S1), which amplify fragments of about 180 bp from internal gene sequences, were used together with cDNA as template in qPCR reactions. *hrdB* was used as housekeeping gene in each experiment in order to standardize the results by eliminating variation in RNA and cDNA quantity and quality. Each reaction mixture of 10 μL volume contained 5 μL SYBR^®^ Green Supermix (BioRad), 3.85 μL nuclease-free water, 0.2 μL of each primer, and 0.75 μL template. PCR conditions were 98°C for 5 min, followed by 35 cycles of 95°C for 20 s, and 57°C for 30 s. To determine amplification specificity, melting curve analyses were performed after the last cycle, showing in all cases one single peak. Results were analyzed using the ΔΔCT-method (Livak and Schmittgen, 2001). Changes in gene expression are represented in relation to the data from samples of *SLpGM190*. Data are presented as the results from six independent biological replicates.

### Database Analysis

FASTA sequences of SARP proteins were extracted from the antiSMASH database version 2 (Blin et al., 2019) by querying for all genes that hit the antiSMASH smCoG (secondary metabolite clusters of orthologous groups) profile SMCOG1041 (transcriptional regulator, SARP family). These hits were checked for the presence of at least one out of four SARP-related profiles from the PFAM database: PF00486.27 (Trans_reg_C, the HTH-style DNA binding domain), PF93704.16 (BTAD, the transcriptional activator), PF00931.22 (NB-ARC, a domain of unknown function found, e.g., in *Saccharopolyspora erythraea* SARPs), and PF13424.6 (TPR_12, a TPR found in many larger SARPs). Sequences that did not hit at least one of these domains were discarded. Sequences were then annotated by which of the four profiles were hit and grouped by taxonomic order and BCG type.

## Results

### PapR2 Induces Expression of the Silent Red Gene Cluster in *S. lividans*

**FIGURE 1:**
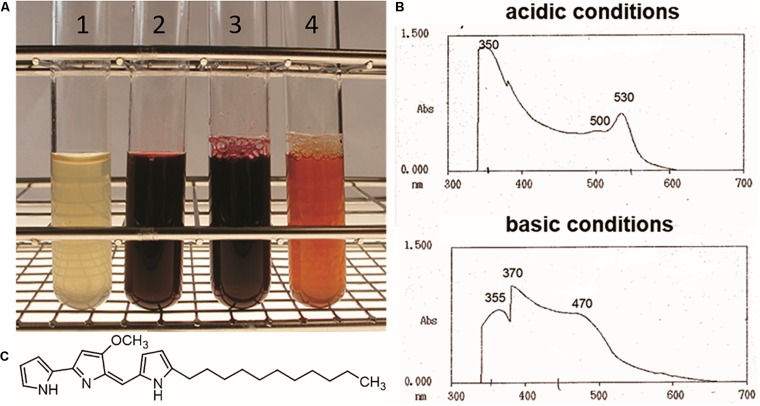
Culture filtrates from *SLpGM190* (1) and *SLpapR2-OE* (2) samples after 3 days of cultivation. Culture filtrate of *SLpapR2-OE* under acidic (3) and basic (4) conditions **(A)**. Absorption profile of the *SLpapR2-OE* culture filtrate under acidic (upper graph) and basic condition (lower graph) **(B)**. Chemical structure of Red **(C)**.

*Streptomyces lividans* is a widely used heterologous host strain, which under specific laboratory conditions does not exhibit production of the two pigmented secondary metabolites actinorhodin and undecylprodigiosin (Hu et al., 2002; Martinez et al., 2005; Rodríguez et al., 2013). In frame of analyzing the regulatory role of PapR2 in *S. pristinaespiralis*, the *papR2* gene was heterologously expressed in *S. lividans* T7 for protein purification purposes (Mast et al., 2015). For overexpression experiments the *S. lividans* strain *SLpapR2-OE* was used, which harbors the *papR2* gene under control of the thiostrepton-inducible promoter P_tipA_ on the replicative medium-copy plasmid pGM190 (Mast et al., 2015). *S. lividans* strain *SLpGM190* was used as a reference, containing the pGM190 empty vector. After 2–3 days of growth in YEME liquid medium with thiostrepton as inductor for gene expression, the whole *SLpapR2-OE* culture, as well as the culture supernatant showed an intensive red pigment formation, which was not observed for samples of *SLpGM190* (Figure 1A). This phenomenon was also observed on R5 agar with thiostrepton, where *SLpapR2-OE* mycelium was intensively red colored after 3–4 days of cultivation, whereas *SLpGM190* mycelium was not (Supplementary Figure S1). *S. lividans* is known to harbor a Red BCG, which remains silent under normal growth conditions (Horinouchi et al., 1986). Red biosynthesis has mainly been studied in *S. coelicolor*; however, since *S. coelicolor* and *S. lividans* are very closely related species, knowledge on Red biosynthesis and regulation can be transferred to *S. lividans* (van Wezel et al., 2000; Lewis et al., 2010). To investigate, whether the red color of the *SLpapR2-OE* cultures originates from the formation of the Red metabolite, we performed spectrophotometrical analysis. A pH shift was carried out with the culture supernatant of *SLpapR2-OE* and the absorption maxima of the sample was determined by using a Hitachi U-2000 spectrophotometer. Spectrophotometrical analysis led to the detection of the Red-specific spectral absorption maxima (Onaka et al., 2002) at 533 and 468 nm under acidic and basic conditions, respectively (Figure 1B), which proved that the *SLpapR2-OE* samples contained Red (Figure 1C). These data suggested that the overexpression of *papR2* in *S. lividans* induced Red biosynthesis.

### PapR2 Mimics the Function of the *S. lividans* SARP-Type Regulator RedD

**FIGURE 2:**
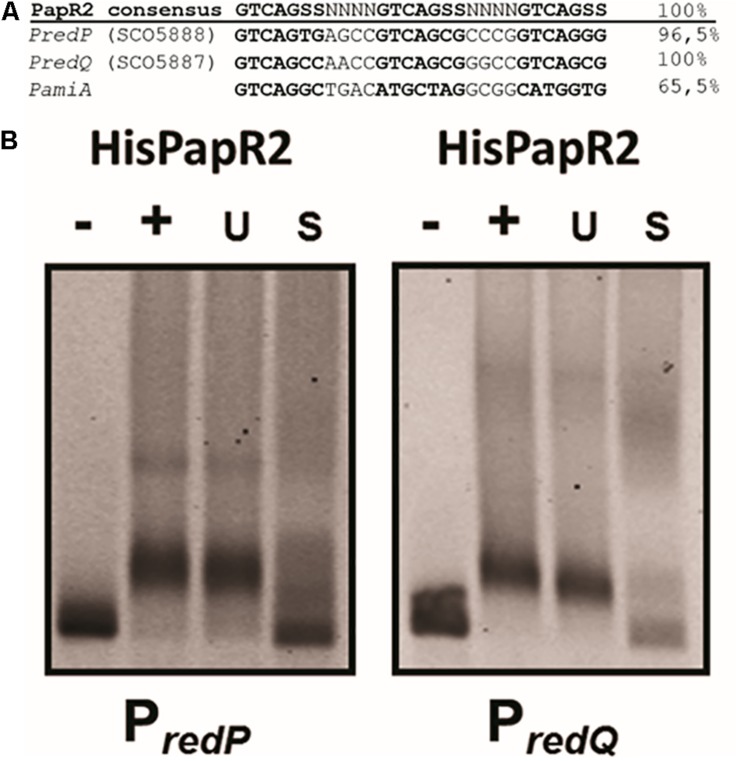
SARP binding sequences within the upstream region of the RED-biosynthetic genes *redP* (SCO5888), *redQ* (SCO5887), and *pliA* **(A)**. EMSAs with undefined HisPapR2 concentrations and Cy5-labeled promoter regions of *redP* and *redQ.* – indicates negative control without protein, + indicates addition of HisPapR2 protein. The specificity of the reaction was checked by the addition of 500-fold specific (S) and unspecific (U) unlabeled DNA **(B)**.

In *S. lividans* Red biosynthesis is under control of the SARP-type regulator RedD, which directly activates the Red biosynthetic genes (Takano et al., 1992; White and Bibb, 1997). An amino acid sequence comparison using BLASTP revealed that PapR2 and RedD are highly similar to each other (44% identity, 55% similarity) (Mast et al., 2015). This amino acid sequence similarity was even higher for the HTH motif of the protein (66% identity, 75% similarity). Thus, we suspected that PapR2 may substitute for the function of RedD and activates Red biosynthesis in *S. lividans.* In order to identify potential SARP-type binding motifs within the *S. lividans* Red BGC, the genome was analyzed with the bioinformatic tool PatScan, which allows for the identification of specific sequence patterns in a given genome sequence (Blin et al., 2018). PatScan analysis was performed with the previously described PapR2 consensus motif (5’-GTCAGSS-3’) (Mast et al., 2015) as sequence pattern on the *S. lividans* genome sequence. Thereby, two highly conserved PapR2-like motifs were identified within the intergenic region of the Red-specific biosynthetic genes *redP* (SCO5888) and *redQ* (SCO5887) with each 100 and 96.5% identity, respectively (Figure 2A). *redP* encodes a 3-ketoacyl-acyl carrier protein synthase, whereas *redQ* codes for an acyl carrier protein, both of which have been shown to be involved in Red biosynthesis (Mo et al., 2008). To analyze the functionality of the identified SARP motifs, EMSAs were performed with the PapR2 protein and the upstream regions of *redP* (P*redP*, 182 bp) and *redQ* (P*redQ*, 182 bp), respectively, harboring the PapR2 consensus sequence. EMSAs showed that PapR2 specifically binds to the P*redP* and P*redQ* fragment, respectively (Figure 2B), revealing the functionality of the identified SARP-type motifs.

**FIGURE 3:**
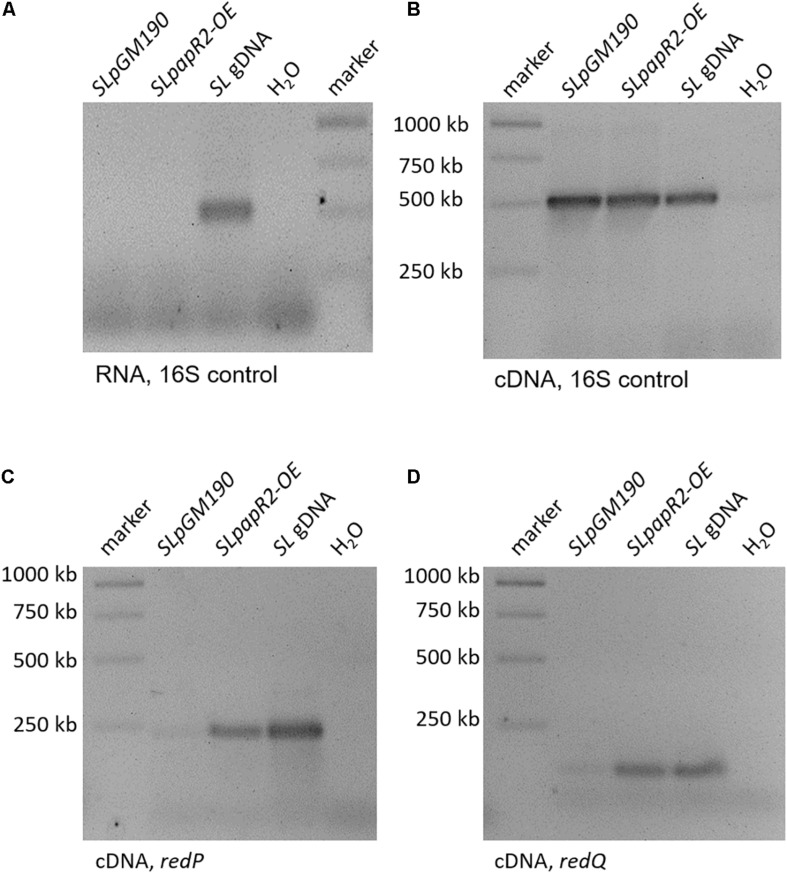
Transcriptional analysis of samples from *SLpGM190* and *SLpapR2-OE* after 168 h. gDNA of *S. lividans* was used as internal positive control (SL gDNA) and water as internal negative control. RT-PCR analysis with RNA samples as template and 16S-specific primers in order to test the absence of DNA **(A)**. RT-PCR analysis with cDNA samples as template and 16S-specific primers in order to test for successful cDNA synthesis **(B)**. RT-PCR analysis of the gene *redP* and in the strains SLpGM190 and SLpapR2-OE **(C)**. RT-PCR analysis of the gene *redQ* and in the strains SLpGM190 and SLpapR2-OE **(D)**.

To confirm the regulatory effect of PapR2 on the transcription of the Red BGC, we performed RT-PCR and quantitative qPCR experiments. For these studies *SLpapR2-OE*, as well as the reference strain *SLpGM190* were grown in R5 medium. After 72 h of cultivation samples were harvested for RNA isolation. For each strain six biological replicates were performed. Isolated RNA was used as a template in RT-PCR experiments as a negative control (Figure 3A), whereas cDNA was used together with 16S primers as a positive control (Figure 3B). For *red* gene-specific transcriptional analysis, isolated RNA was used as template for RT-PCR experiments with the primer pairs redPintfw/rv and redQintfw/rv, which annealed to internal parts of *redP* and *redQ*, respectively. The transcriptional analysis revealed that there are stronger signals for the *redP* and *redQ* cDNA amplificates in *SLpapR2-OE* samples compared to *SLpGM190* samples (Figures 3C,D, respectively). In order to quantify the amount of *redP* and *redQ* RT-qPCR was performed. Based on the threshold cycle it was calculated that *redP* and *redQ* transcripts were increased to 57- and 492-fold, respectively, in samples of *SLpapR2-OE* compared to samples of *SLpGM190* (Supplementary Figure S2). Thus, transcriptional analyses demonstrated that PapR2 activates the transcription of the Red biosynthetic genes *redP* and *redQ*. Due to the intensive Red production of strain *SLpapR2-OE* together with the data obtained from PapR2 EMSA studies and transcriptional analysis, we propose that overexpressed PapR2 in *S. lividans* takes over the regulatory function of RedD and activates the transcription of the Red biosynthetic genes, which leads to the formation of the red colored secondary metabolite Red.

### PapR2 Expression Improves Antibiotic Activity of SHP22-7

**FIGURE 4:**
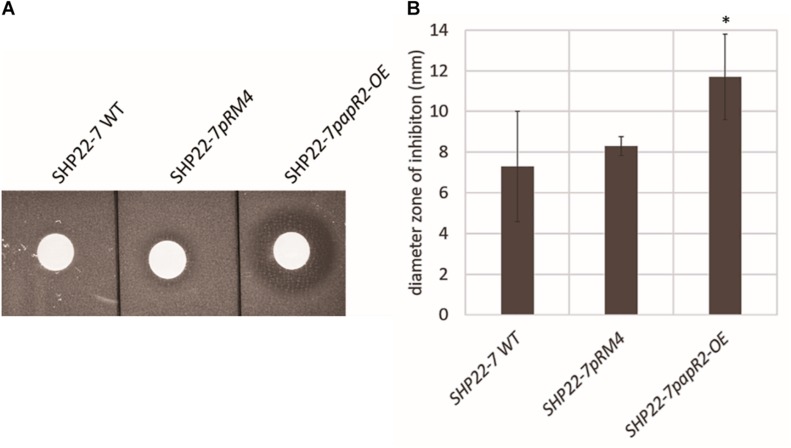
One representative example of culture extracts from SHP22-7 WT, SHP22-7*pRM4*, and SHP22-7*papR2-OE*, respectively, against *B. subtilis.* Filter disk diffusion assay; red arrow indicates inhibition zone **(A)**. Graphical representation of the inhibition zone diameters (mm) from 10 independent biological replicates (*n* = 10), ^∗^ indicates significance **(B)**.

In order to study the general application of SARP-type regulators for activation of antibiotic biosynthesis, we exemplary tested strain SHP22-7 as a host for *papR2* expression. SHP22-7 is a novel strain isolate from a soil sample of the unique desert island Enggano, Indonesia (Handayani et al., 2018). SHP22-7 shows broad-spectrum antibacterial activity against Gram-positive and Gram-negative bacteria, including *E. coli*, *Staphylococcus carnosus*, *Micrococcus luteus*, and *B. subtilis*. The SHP22-7 genome has been sequenced recently and antiSMASH analysis led to the identification of 25 potential secondary metabolite gene clusters (Handayani et al., 2018; Table 1). Four of the gene clusters (clusters 3, 6, 10, and 15) contain SARP genes, which makes SHP22-7 a good candidate strain for a SARP-guided activation. For *papR2* expression studies in SHP22-7, the *papR2* gene was cloned into the integrative plasmid pRM4 under control of the constitutive *ermE*^∗^ promoter, resulting in construct pRM4/papR2. The pRM4 vector was used as expression plasmid in these analyses to avoid addition of thiostrepton as inductor (see above), which would influence subsequent antibacterial bioassays. The plasmid was transferred to SHP22-7 by protoplast transformation. The resulting expression strain SHP22-7*papR2-OE*, as well as the two reference strains SHP22-7*pRM4*, which harbors the empty pRM4 vector, and the SHP22-7 wild-type (WT) were used for antibacterial bioassay studies. SHP22-7*papR2-OE*, SHP22-7pRM4, and SHP22-7 WT were each cultivated in NL19 medium and samples were taken at 168 h. Methanolic culture extracts were applied for antibacterial bioassays using *B. subtilis* as test organism. Filter discs with kanamycin and methanol served as positive and negative control, respectively. Bioassays were carried out as 10 independent biological replicates. Thereby, significantly larger inhibition zones against *B. subtilis* were observed on average with extracts from SHP22-7*papR2-OE* compared to extracts from SHP22-7*pRM4* or SHP22-7 WT (Figure 4A). The extract of SHP22-7*papR2-OE* caused a zone of inhibition of 11.7 ± 2.1 mm, whereas the extracts of SHP22-7*pRM4* and SHP22-7 WT yielded smaller inhibitions zones of 8.3 ± 2.6 and 7.3 ± 2.7 mm, respectively (Figure 4B). These data showed that *papR2* expression in SHP22-7 leads to a significantly improved antibiotic activity.

**TABLE 1:** Secondary metabolite gene clusters of *Streptomyces* sp. SHP22-7 as predicted by antiSMASH 4.0 with an indication of the presence of cluster-situated SARP genes (count), predicted SARP proteins with amino acid sequence homology to PapR2 (I = identity, S = similarity), as well as identified SARP-type motifs.

Cluster	Type of secondary metabolite gene cluster	Similarity to known cluster	Localization	Cluster-situated SARP gene	Sequence homology to PapR2I/S	SARP consensus motif(s) identified
1	Terpene	Albaflavenone (100%)	Contig 1 49588-70841	−		−
2	T2PKS	Spore pigment (66%)	Contig 1 121312-163740	−		−
3	Other	Granaticin (8%)	Contig 2 304806-348126	+ (1)	41/52%	+
4	Melanin	Melanin (60%)	Contig 3 1-5936	−		−
5	Ectoine	Ectoine (100%)	Contig 4 119873-130271	−		−
6	NRPS	Phosphonoglycans (5%)	Contig 5 129226-170626	+ (1)	31/44%	+
7	Bacteriocin	−	Contig 7 213837-225168	−		−
8	Terpene	−	Contig 7 240589-262769	−		−
9	Other	Amicetin (75%)	Contig 10 129625-170245	−		+
10	NRPS	Calcium dependent antibiotic (47%)	Contig 10 243082-272180	+ (1)	38/56%	+
11	T3PKS	Herboxidiene (8%)	Contig 11 20911-62017	−		−
12	Siderophore	Desferrioxamine B (100%)	Contig 13 127488-139260	−		−
13	Indole	Antimycin (20%)	Contig 17 55086-76126	−		−
14	Terpene	Carotenoid (36%)	Contig 17 133951-147999	−		−
15	T2PKS-Butyrolactone	Fluostatin (26%)	Contig 19 32007-96281	+ (3)	41/59% 67/76% 40/51%	+
16	Terpene	Hopene (84%)	Contig 20 8526-35239	−		−
17	Lanthipeptide	−	Contig 21 82417-98812	−		−
18	T1PKS-NRPS	Candicidin (90%)	Contig 22 1-86799	−		−
19	Arylpolyene-NRPS	Lipopeptide (29%)	Contig 24 1-40688	−		−
20	Siderophore	Grincamycin (8%)	Contig 24 46763-57062	−		−
21	T1PKS	Oligomycin (44%)	Contig 32 4049-49007	−		−
22	OtherKS	Sanglifehrin A (13%)	Contig 42 1-35910	−		−
23	Bacteriocin	Informatipeptin (57%)	Contig 44 22689-32904	−		−
24	NRPS	Coelichelin (27%)	Contig 59 1-16803	−		−
25	Lanthipeptide	−	Contig 96 1-5597	−		−

*+, cluster contains gene/motif; −, cluster does not contain gene/motif.*

### PapR2 Activates Transcription of Cluster 9 in SHP22-7

**FIGURE 5:**
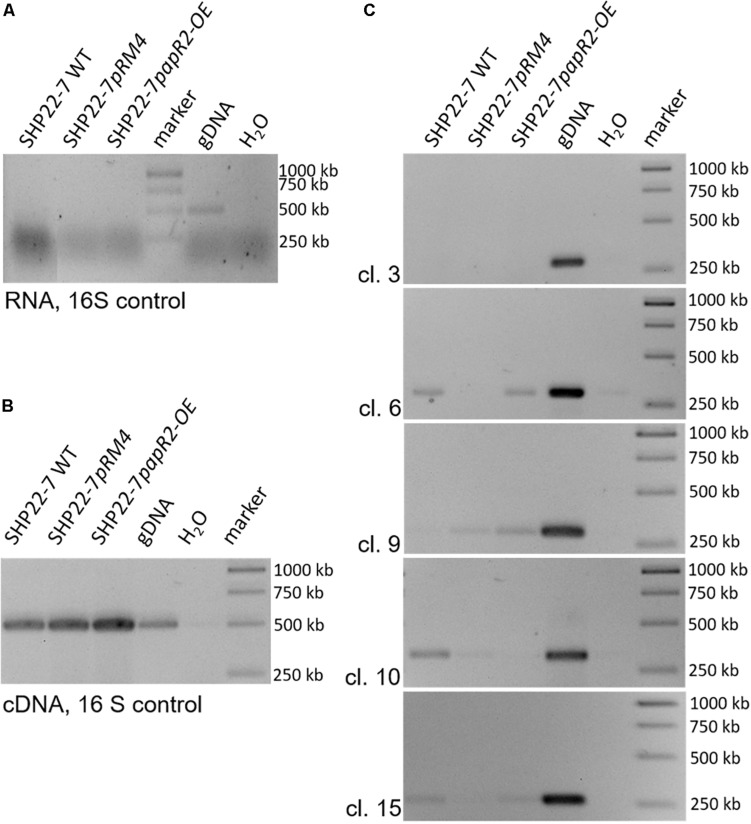
Transcriptional analysis of the SHP22-7 strains SHP22-7 WT, SHP22-7pRM4, and SHP22-7papR2; gDNA of SHP22-7 was used as internal positive (gDNA) and water as internal negative control. The data are representative for four independent experiments. RT-PCR analysis with RNA as template and 16S-specific primers **(A)**. RT-PCR analysis with cDNA and 16S-specific primers **(B)**. Transcriptional analysis of the SARP motif containing gene clusters (cl.) 3, 6, 9, 10, and 15 with samples from SHP22-7 WT, SHP22-7pRM4, and SHP22-7papR2 **(C)**.

To identify the substance that is responsible for the antibiotic activity of SHP22-7 but avoid elaborative chemical analytics in the first place, we screened the SHP22-7 genome sequence for the occurrence of PapR2-like consensus sequences by using the PatScan tool. Here, we only considered motifs that were located within intergenic regions of genes from suggested SHP22-7 BGCs. The motif search led to the identification of PapR2-like consensus sequences within 5 of the 25 predicted BGCs from SHP22-7. These included clusters 6 and 10 (NRPS-like gene cluster), cluster 15 (type 2 PKS-butyrolactone-like gene cluster), as well as clusters 3 and 9 (“other” type of gene cluster) (Table 1). In order to find out which of the five gene clusters is activated by PapR2, comparative RT-PCR analysis was carried out with SHP22-7*papR2-OE*, SHP22-7pRM4, and SHP22-7 WT in four independent biological replicates. Cells were grown under antibiotic production conditions and samples were harvested at 168 h of cultivation. Two samples were collected for each strain and growth time point, whereby one sample was used for RT-PCR analysis and the second one for HPLC–MS analysis (see the section “SARP-Type Regulatory Genes Are Widespread in BGCs From Diverse Actinobacteria”). RT-PCR was carried out with RNA isolated from cell pellets of SHP22-7*papR2-OE*, SHP22-7*pRM4*, and SHP22-7 WT, respectively. Isolated RNA was used as a template in RT-PCR experiments as a negative control (Figure 5A), whereas cDNA was used together with *16S* primers as a positive control (Figure 5B). For cluster specific transcriptional analysis, cDNA was generated with primer pairs cl3fw/rv, cl6fw/rv, cl9fw/rv, cl10fw/rv, and cl15fw/rv (Supplementary Table S1), each aligning to a predicted biosynthesis gene of clusters 3, 6, 9, 10, and 15, respectively. The transcriptional analysis revealed that there is a stronger signal for the cluster 9 amplificate in the SHP22-7*papR2-OE* samples compared to the samples of SHP22-7*pRM4* and SHP22-7 WT, where there is nearly no signal at all (Figure 5C). This difference in signal intensity could not be observed for clusters 3, 6, 10, and 15 (Figure 5C). Thus, these data suggested that PapR2 activates the transcription of cluster 9 in SHP22-7*papR2-OE.*

### Cluster 9 Resembles an Amicetin Gene Cluster

**FIGURE 6:**
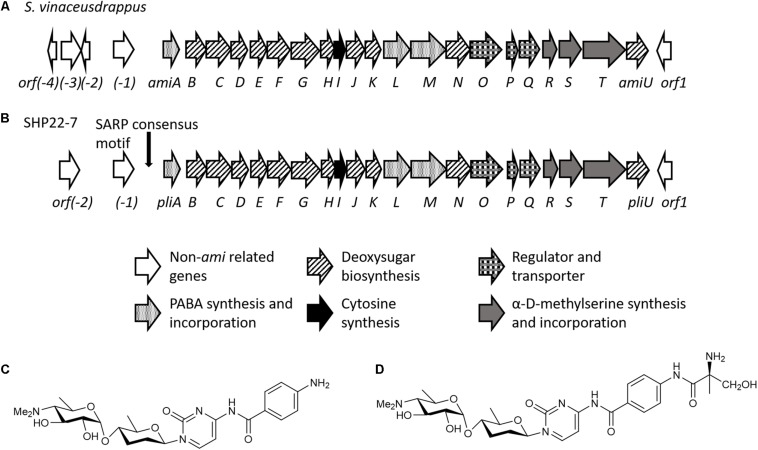
Schematic organization of the amicetin biosynthetic gene cluster of *S. vinaceusdrappus* according to Zhang G.G. et al. (2012) **(A)** and of SHP22-7 **(B)**. Chemical structures of plicacetin **(C)** and amicetin **(D)**.

In order to deduce for what type of metabolite cluster 9 encodes for, we analyzed the cluster sequence region in detail. The antiSMASH output predicted a 75% similarity of cluster 9 to the plicacetin/amicetin gene cluster from *Streptomyces vinaceusdrappus* NRRL 2363 (Table 1). The cluster similarity describes the number of genes with a similarity above the ClusterBlast threshold of 30% sequence similarity at over 25% coverage. Manually cluster analysis by sequence alignments yielded a 100% sequence similarity since the sequence region from *amiA–amiD* was not recognized by antiSMASH to be part of the amicetin BGC (Figures 6A vs. B). Plicacetin (Figure 6C) and amicetin (Figure 6D) are disaccharide pyrimidine nucleoside antibiotics with a broad-spectrum antibacterial (especially against *Mycobacterium tuberculosis*) and antiviral activity. They act as peptidyl transferase inhibitors and thus inhibit protein synthesis. Amicetin consists of the two deoxysugar moieties, D-amosamine and D-amicetose, as well as cytosine, *p*-aminobenzoic acid (PABA), and a terminal methylserine moiety, whereby the latter moiety is missing in plicacetin (Zhang G.G. et al., 2012; Korzybski et al., 2013). The gene cluster analysis of SHP22-7 revealed that the PapR2-like motif is located directly in front of the *orf pliA* (Figure 6B) and shows a rather weak sequence identity of 65.5% to the PapR2 consensus motif (Figure 2A). The same motif is present upstream of *amiA* in *S. vinaceusdrappus.* The *pliA* gene presents 99.75% gene nucleotide sequence identity to *amiA* of *S. vinaceusdrappus*, which translates to a 100% amino acid sequence identity among the predicted gene products. In *S. vinaceusdrappus amiA* is the first gene of the amicetin BCG and encodes a putative 4-amino-4-deoxychorismate lyase, which is suggested to catalyze the conversion from 4-amino-4-deoxychorismate to PABA (Zhang G.G. et al., 2012). Remarkably, all genes in the amicetin gene cluster of *S. vinaceusdrappus* and the amicetin-like gene cluster of SHP22-7 are organized in one direction, suggesting a unidirectional transcription. In this context, it would make sense that regulatory activation targets the promoter of the first gene of the unidirectional BGC. In order to find out if PapR2 can bind to the *pliA* promoter region, EMSAs were performed with the PapR2 protein and the *pliA* upstream region containing the PapR2 consensus sequence. However, no shifted band was detected in these assays (data not shown). Thus, it might be that the motif is not functional at all and does not constitute a SARP binding motif. It could also be that due to the less conserved PapR2 consensus sequence, the motif is not functional in such an *in vitro* assay. The presence of a SARP consensus sequence would hint for a pathway-specific regulatory gene located within the amicetin(-like) BGC. However, a SARP-type regulatory gene has not been identified in any of the amicetin(-like) clusters from SHP22-7 nor *S. vinaceusdrappus* (Table 1; Zhang X. et al., 2012). Overall, six SARP genes have been identified in total within the SHP22-7 genome (Supplementary Table S2). All of them are part of BGCs (clusters 3, 6, 10, and 15). Cluster 15 harbors three SARP genes, whereas the other BGCs each contain one SARP gene (Table 1). Furthermore, putative SARP binding motifs have been found within the promoter regions of the BGCs (Table 1 and Supplementary Table S2). It might be possible that one of these SARP-type regulators plays a role in *trans*-activating amicetin cluster transcription. So far, regulation of amicetin biosynthesis is not understood. In *S. vinaceusdrappus* three genes [*orf*(*-3*), *orf*(*-2*), *amiP*] have been identified, which encode for putative transcriptional regulators, whereby *orf*(*-3*) and *orf*(*-2*) seem not to be part of the amicetin BGC and *amiP* codes for TetR-like transcriptional regulator, which usually function as repressors of antibiotic biosynthesis (Zhang G.G. et al., 2012). Thus far, it cannot be excluded that also *trans*-acting regulator(s) are involved in the regulation of amicetin biosynthesis.

### PapR2 Activates Plicacetin Production in SHP22-7

**FIGURE 7:**
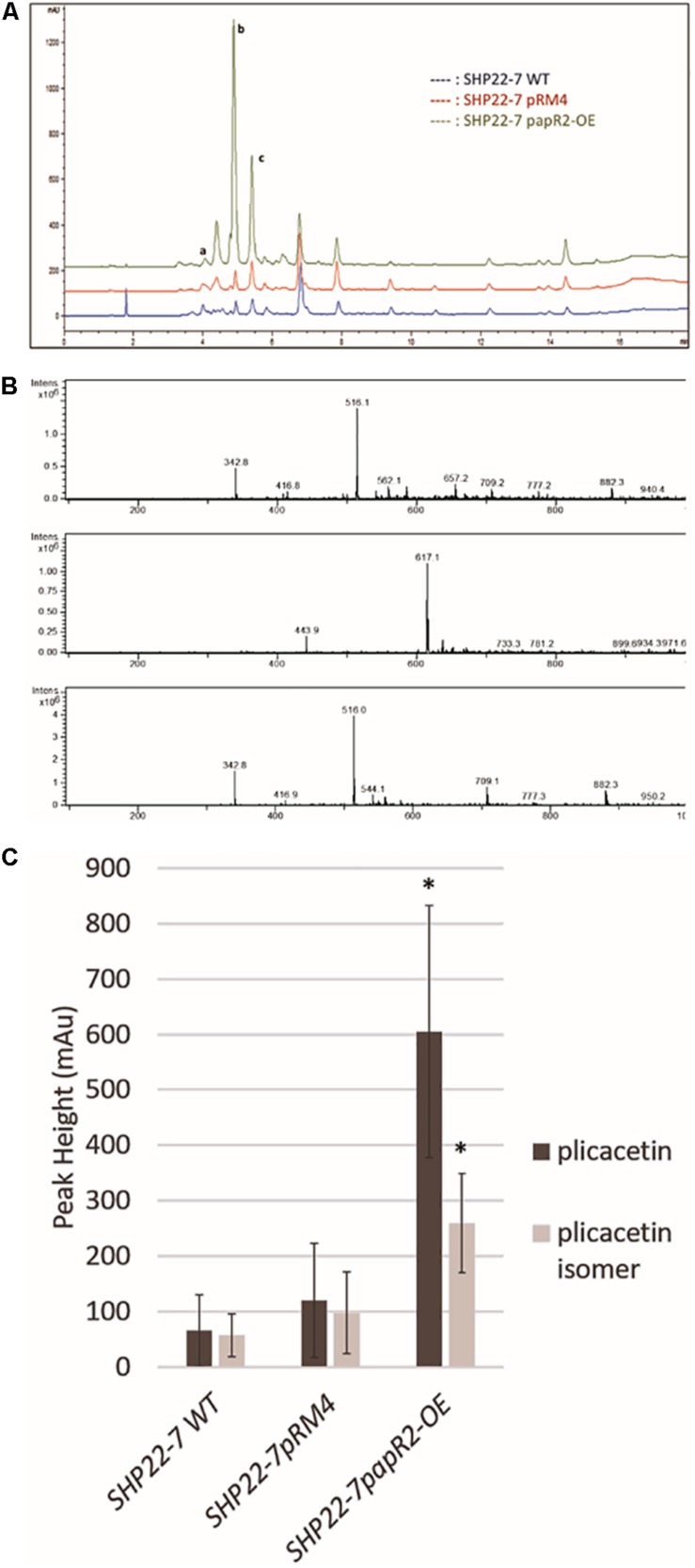
Plicacetin and plicacetin isomer production in the strains SHP22-7 WT, SHP22-7*pRM4*, and SHP22-7*papR2-OE*, respectively. HPLC profile of all three strains. Peaks in the HPLC spectrum representing amicetin are marked with a, plicacetin marked with b, and plicacetin isomer marked with c **(A)**. Mass spectra of plicacetin **(Upper)**, amicetin **(Middle)**, and a plicacetin isomer **(Lower)(B)** isolated from SHP22-7 WT. Graphical representation of production levels of plicacetin and plicacetin isomer in each strain. *n* = 10; ^∗^ means significance **(C)**.

To investigate if an amicetin(-like) antibiotic is produced by SHP22-7, we analyzed methanolic extracts of cell culture samples from SHP22-7*papR2-OE* by HPLC/MS. To analyze if production is influenced by *papR2* expression, extracts from samples of SHP22-7*pRM4* and SHP22-7 WT served as references. For these analyses we used the second culture samples obtained from SHP22-7*papR2-OE*, SHP22-7*pRM4*, and SHP22-7 WT, respectively (see above). Based on comparisons with an in-house substance database, HPLC analysis revealed the presence of amicetin [retention time (RT) 4.0 min], plicacetin (RT 4.8 min), and a plicacetin isomer (RT 5.6 min) in all three samples (Figure 7A). The identity of the compounds was verified by MS/MS analysis (amicetin *m/z* 617.1 [M−H]; plicacetin *m/z* 516.1 [M−H]; plicacetin isomer *m/z* 516.1 [M−H]) (Figure 7B; for HRMS data see Supplementary Figures S3, S4). Comparisons between the HPLC spectra from samples of SHP22-7*papR2-OE*, SHP22-7*pRM4*, and SHP22-7 WT displayed that peak intensities were especially increased for plicacetin (mAU 605) and the plicacetin isomer (mAU 259) in samples of SHP22-7*papR2-OE* compared to samples of SHP22-7pRM4 [plicacetin (mAU 120), plicacetin isomer (mAU 97)] and SHP22-7 WT [plicacetin (mAU 66), plicacetin isomer (mAU 57)] (Figure 7C). Thus, it could be shown that *papR2* expression in SHP22-7 activates explicitly plicacetin biosynthesis, whereas amicetin biosynthesis seems not to be affected. Overall, with this result it could be confirmed that PapR2 induced cluster 9 transcription, which resulted in an increased plicacetin production. Thus, it can be concluded that the increased bioactivity of SHP22-7*papR2-OE* samples against *B. subtilis* arises from the increased production of the nucleoside antibiotic plicacetin. However, since no direct interaction of the PapR2 regulator with the amicetin promoter region could be shown by EMSA analysis, it cannot be deduced if the activation effect is a direct or an indirect one. The SARP consensus motif harbors the central TCA triad, which is also present in the Pho box (GTTCACC), resembling the target site of the phosphate control two-component system PhoP/PhoR (Martin and Liras, 2020). This sequence region is also known to be bound by the large size SARP regulator AfsR (Martin and Liras, 2020). Additionally, the central TCA triad can be present in binding motifs recognized by the nitrogen regulator GlnR or the DmdR1 (Flores and Martín, 2004; Martin and Liras, 2020). Cross-talk between different transcriptional regulators via the interaction of the same binding sites have been shown before (Martín et al., 2011; Martin and Liras, 2020). Thus, it is also possible that transcriptional activation of the amicetin BGC is an effect from multiple regulatory interactions.

### SARP-Type Regulatory Genes Are Widespread in BGCs From Diverse Actinobacteria

Of the top 10 genera containing SARP-type regulators in the antiSMASH database 2 (Blin et al., 2019), 9 belong to the phylum Actinobacteria. Broken up by genus, 98% of *Streptomyces* (611/625), 81% of *Nocardia* (78/96), 100% of *Salinispora* (72/72), 100% of *Micromonospora* (62/62), and 97% of *Amycolatopsis* (38/39) genomes harbor SARP-type regulatory genes, showing their prevalence in filamentous Actinobacteria. On the other hand, only 42% of *Mycobacterium* (115/276) species have SARP-type regulators. Outside of Actinobacteria, mainly Proteobacteria have hits for the SMCOG1041 profile, but mostly lack hits for the HTH motif domain or the BTAD (transcriptional activator domain) [e.g., 6% (79/1236) of *Pseudomonas* hit the smCoG profile, but none of them contain a BTAD match]. Thus, SMCOG1041 profiles from Proteobacteria may not represent typical SARP-type regulators.

**FIGURE 8:**
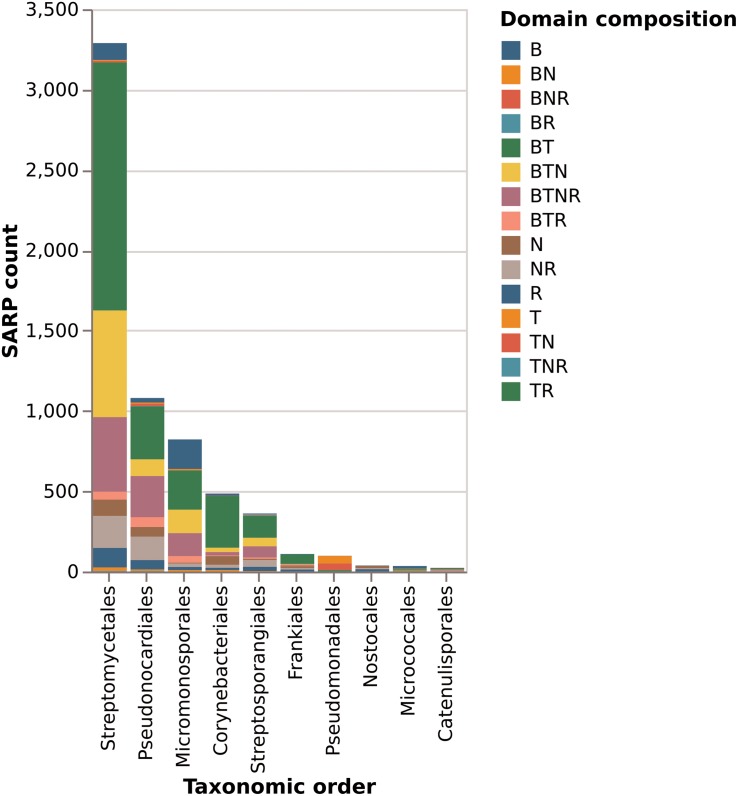
Domain composition of SARP-like proteins in the 10 orders with most protein hits. Letters in the legend denote which domains are present. B is the BTAD domain, T is the HTH-motif DNA binding domain, N is the NB-ARC domain of unknown function, and R is the tetratricopeptide repeat domain.

**FIGURE 9:**
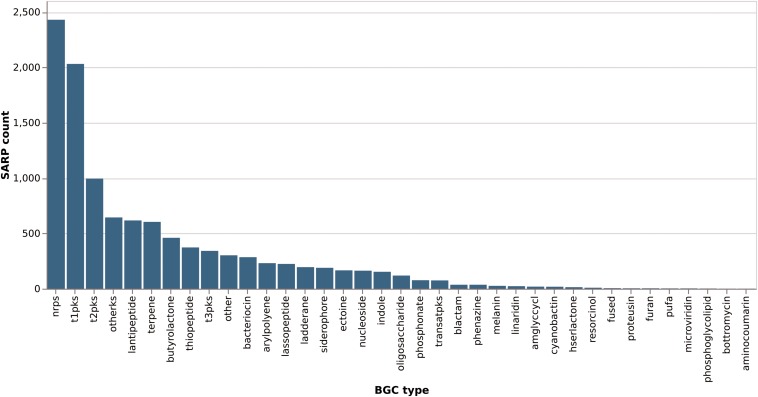
Distribution of SARP-like genes across different biosynthetic gene cluster types. SARP-like genes in hybrid clusters are counted once per type they occur in (e.g., a SARP in a NRPS/PKS type I hybrid cluster would count for both “nrps” and “t1pks”).

Of the total of 6525 proteins containing a hit against any of the four SARP-related PFAM domains, 3289 (50%) are from the order *Streptomycetales* (Figure 8). These again break down into 47% “small” SARPs (only containing the HTH and BTAD domains) and 36% “large” SARPs (also containing the NB-ARC and/or TPR domains). The remaining proteins miss the HTH and/or BTAD domains, likely an artifact of bad sequencing data in published draft genomes. The overrepresentation of SARP-type regulators in *Streptomycetales* may also be explained by the higher abundance of available genomes in the database. *Pseudonocardiales* cover 17% of the SARP-type proteins (1080/6525). 31% (330/1080) are “small” SARPs and 39% (421/1080) “large” SARPs. In *Micromonosporales*, accounting for 13% (822/6526) of the dataset, 30% (244/822) are “small” SARPs, and 40% (330/822) are “large” SARPs (Figure 8). Interestingly, *Micromonosporales* is the only order to contain a significant amount (22%, 184/882) of SARP-type proteins only containing a hit against BTAD without hitting the HTH domain, suggesting a different subfamily of transcriptional activator. Furthermore, SARP genes were found to be present in various different types of BGCs with a prominent abundance in NRPS and PKS gene clusters (Figure 9), which however might also be associated with the higher frequency of these cluster types in the database.

## Discussion

Regulation is the screw plug to unlock the biosynthetic potential of natural compound producers. Here, we showed that heterologous expression of the SARP-type regulatory gene *papR2* in the foreign host *S. lividans* leads to transcriptional activation of the silent Red BGC. Since SARP regulators show a comparable protein architecture and bind to similar recognition sequences at the DNA, they can substitute for their regulatory functions as exemplified for PapR2 and RedD. This is also underpinned by a previous study, where it has been shown that overexpression of the SARP gene *vlml* from the valanimycin producer *Streptomyces viridifaciens* in a *redD* mutant of *S. coelicolor* M512 restores Red production, demonstrating that *vlmI* can complement a *redD* mutation (Garg and Parry, 2010). Besides having gained indications for such kind of cross-regulation, our data also contribute to a better understanding of the regulation of Red biosynthesis in general. So far, there have been only bioinformatic predictions on potential regulatory binding regions within the Red BGC (Iqbal et al., 2012). Here, we provide first experimental evidence by EMSA studies and (q)RT-PCR experiments for the SARP-binding capability to promoter regions (P*redP*, P*redQ*) of the Red BGC. These data clearly show that *redP* and *redQ* are direct targets of a SARP-driven regulation during Red biosynthesis.

In the literature SARPs often are designated as “pathway-specific” transcriptional activators, which means that they control the expression of an individual BCG. However, SARPs indeed can have activating effects on the biosynthesis of different substances. One example occurring in nature is provided by the SARP-type regulator CcaR from *S. clavuligerus*, which activates the cephamycin gene cluster, where *ccaR* is part of, as well as the adjacent clavulanic acid gene cluster (Pérez-Llarena et al., 1997). Thus, the term “pathway-specific” is not accurate for SARPs and should better be replaced by “cluster-situated” as suggested previously (Huang et al., 2005; Liu et al., 2013). Our results show that SARPs have the potential to control different BGCs, when present in different producer organisms: The SARP regulator PapR2 activates the transcription of the corresponding pristinamycin gene cluster in *S. pristinaespiralis* (Mast et al., 2015) but it also affects different antibiotic BGCs, such as Red and plicacetin, when expressed in foreign strains, such as *S. lividans* or SHP22-7, respectively. Especially, in SHP22-7 *papR2* expression significantly improved plicacetin production and as a result also led to an improved production of a so far not further characterized plicacetin derivative. This derivative is dissimilar from the plicacetin isomer since it shows a different retention time in HPLC analysis (∼6.5 min) (Figure 9) and a smaller mass of 481 *m*/*z* (data not shown). If PapR2-driven plicacetin gene cluster activation is a direct or an indirect regulatory effect is unclear at the moment since no direct interaction of the PapR2 regulator with the plicacetin gene cluster could be demonstrated. Furthermore, no SARP gene is present in the plicacetin gene cluster. However, SARP genes have been found in four other SHP22-7 BGCs, which would allow the possibility that one of these regulators may act as the natural activator of amicetin/plicacetin biosynthesis. A similar untargeted effect has been observed when the PAS-LuxR-type regulator PimM from *S. natalensis* was expressed in *S. clavuligerus*, which led to an improved production of cephamycin, clavulanic acid, and tunicamycins (Martínez-Burgo et al., 2019). For tunicamycin production the regulatory effect upon *pimM* expression is unclear. *pimM* expression improved tunicamycin production without affecting tunicamycin gene cluster transcription. Besides, no pathway-specific activator has been identified within the tunicamycin BGC. In this study, the authors speculated that PimM may exert its effect on tunicamycin production, e.g., due to a positive influence on precursor supply (Martínez-Burgo et al., 2019). Furthermore, they found that PimM from *S. natalensis* shows some similarity to a PimM-like regulator, encoded by a gene of the *S. clavuligerus* genome (Martínez-Burgo et al., 2019). Thus, also in these analyses the non-native PimM regulator may have occupied the regulatory role of a homologous natural regulator and along this path provoked antibiotic production. Interspecies cross-regulation has also been shown for the LuxR-family type (LAL) regulator PikD. In *Streptomyces. venezuelae* PikD regulates the expression of pikromycin. The heterologous expression of the *pikD* homologous genes *rapH* and *fkbN* from *Streptomyces hygroscopicus* in *S. venezuelae* increased the production of the antibiotic pikromycin. LAL-family regulators resemble SARP-type regulators in the ATP-binding motif at the N-terminus and in the DNA-binding motif to some extent, which in both regulator types consists of an HTH-motif (Mo and Yoon, 2016).

The fact that SARP genes are widely distributed among actinomycetes, where they occur in many different types of secondary metabolite gene clusters makes them good candidates to be used as engineering tools for the activation of BCGs. Especially in *Streptomyces* they are predominant occurring with a ∼100% abundance. Interestingly, there might be a phylogenetic grouping of different SARP regulator types from different actinobacterial genera. If so this would raise the question if there is a co-evolution of certain regulator genes with their corresponding gene clusters. Besides that, the statistical analysis revealed that there is a different abundance of different types of SARP regulators, belonging either to the small SARPs (<400 amino acids) with only HTH and BTAD domains (e.g., ActII-ORF4 or RedD) or the large SARPs, which contain an additional NTPase domain and/or a conserved C-terminal TPR domain of unknown function (∼1000 residues) (Liu et al., 2013). Overall small SARP type regulators are more abundant in actinomycetes than the large ones (Figure 8). Besides, more experimental data are available on small SARP-guided regulations, and the unclear function of the additional domains included in the large SARPs likely causes further constraints in the function of the regulatory activity. Thus, we propose that especially representatives from the group of small SARP regulators are good candidates to be used for activation approaches. As outlined above, SARP regulators can bind to recognition sequences, which occur at various positions within the promoter of the target genes. The variety of binding sites may also reflect the diversity of SARP-type activators. Thus, it would be interesting to bioinformatically group the SARP regulators by taking into account their DNA-binding domains, which may provide a better picture of the different SARP subsets.

That SARP expression can lead to the activation of silent gene cluster expression has been shown in a recent study. Here, the aim was to activate some of the more than 20 cryptic gene clusters from *Streptomyces* sp. MSC090213JE08 (Du et al., 2016). In this study the authors combined an OSMAC approach with the expression of several native SARP genes from *Streptomyces* sp. MSC090213JE08. Thereby, four of the seven generated recombinant SARP expression strains produced nine metabolites that were hardly detected in the control strains. Expression of one of the SARP genes (SARP-7) in *Streptomyces* sp. MSC090213JE08 led to the production of the novel polyene-like substance ishigamide (Du et al., 2016). This study successfully showed the potency of a SARP-guided silent gene cluster activation. However, the drawback of this approach is that several conditions (number of different culture media) need to be tested and a set of genetic manipulations (cloning of each individual SARP gene) has to be done in order to provoke cluster activation. Further experimental setup is then linked to untargeted laborious compound purifications and analytics. Indeed, this is also the main problem of several other efforts to activate secondary metabolite synthesis in actinomycetes as outlined above. Thus, based on our gained knowledge we propose a targeted SARP-guided strategy for the activation of BGCs in actinomycetes. Our strategy involves (1) prioritization of strains with SARP genes and SARP binding motifs in the BGC. In our experimental setup we focus on SARP genes that encode for predicted proteins with high similarity to PapR2 (>55% amino acid sequence similarity), as well as the occurrence of a PapR2 consensus motif within the promoter region(s) of the SARP-gene containing BGCs, (2) introduction of a SARP-expression construct [in our approach this means heterologous expression of the PapR2 regulator with the help of the pGM190/papR2 and/or pRM4/papR2 expression construct(s)], (3) comparative biological and chemical analyses of SARP-activated expression samples with samples from non-manipulated strains. In our study we focus on PapR2 as the activator brick, however, of course any other type of SARP regulator with a known consensus sequence might be used as the basis for such an activation approach. For sure there might be limitations in such an activation strategy, e.g., transcriptional activation may fail due to SARP-specificity reasons or a lack of a broader set of well-characterized SARP regulators to be tested as candidate elicitors. However, here we disclose a screening idea or a kind of dragnet investigation, which does not aim to cover all possible SARP-regulated clusters but highlights the most probable ones to be activated upon SARP expression. Our strategy has two main advantages: (1) In contrast to completely unspecific cluster activation efforts, such as addition of general elicitors, co-culture approaches or the OSMAC strategy, our approach is more targeted, as it focuses only on a defined set of BGCs, namely those ones that are bioinformatically predicted to be under SARP control. Applying combinatory bioinformatics, such as antiSMASH and PatScan allows to directly identify the associated BGC. In addition, the detection of the activated compound is more straightforward if the outline of the structure can be deduced from the cluster sequence. (2) In contrast to activation strategies that are absolutely specific for the respective BGC, e.g., heterologous expression of the BGC, introduction of artificial promoters in front of the BGC, or the manipulation of cluster-situated regulators, no major effort to manipulate the genome is necessary with our procedure since it only involves cloning of one SARP gene-containing expression construct into the respective strain(s) of interest. These major benefits make the activation of BGCs by SARPs a promising strategy to be applied on putative antibiotic producers.

## Data Availability Statement

The complete genome sequence of *S. lividans* T7 has been deposited at DDBJ/ENA/GenBank under the accession number ACEY00000000. The main genome scaffold sequence of SHP22-7 has been deposited at DDBJ/ENA/GenBank under the accession number QXMM00000000. Raw sequencing data are available under SRA accession number PRJNA489221.

## Author Contributions

YM generated the strains *SLpGM190* and *SLpapR2-OE*, carried out the bioinformatic analyses, and designed, supervised, and coordinated the study. KB performed the statistical analyses. JK created the vector pRM4/papR2 while the vector was inserted in SHP22-7 by IH. IH tested the bioactivity. JK performed all qualitative and quantitative transcriptional analyses. AK and IH performed the HPLC analyses. YM, JK, IH, and KB wrote the manuscript.

## Conflict of Interest

The authors declare that the research was conducted in the absence of any commercial or financial relationships that could be construed as a potential conflict of interest. The reviewer JA declared a past co-authorship with one of the authors AK to the handling Editor.
