# Supplementary material for: Disclosing the Potential of the SARP-Type Regulator PapR2 for the Activation of Antibiotic Gene Clusters in Streptomycetes
Source: Front Microbiol. 2020 Feb 18;11:225. doi: 10.3389/fmicb.2020.00225 (PMC7040171; doi:10.3389/fmicb.2020.00225)
Supplement: Supplementary file 1 [file Data_Sheet_1.docx]

Supplementary Material

Supplementary Table S1: Bacterial strains, plasmids and primers.

| Bacterial strain | | **Description** | **Source or reference** | |
| --- | --- | --- | --- | --- |
| *E. coli* NovaBlue | | *endA1, hsdR17 (r_K12_^–^ m_K12_^+^), supE44, thi-1, recA1, gyrA96, relA1, lac F′[proA^+^B^+,^ lacI^q^ZΔM15::Tn10], (tsr)* | Novagen | |
| *S. lividans* | |  |  | |
| T7 | | *tsr,* T7 RNA polymerase gene | (Fischer 1996) | |
| *SLpGM190* | | *S. lividans*/pGM190 | (Mast et al. 2015) | |
| *SLpapR2-OE* | | *S. lividans*/pGM190/papR2 | (Mast et al. 2015) | |
| SHP22-7 WT  SHP22-7*pRM4* | | Wild-type | (Handayani et al. 2018) | |
| SHP22-7*papR2-OE* | | SHP22-7/pRM4  SHP22-7/pRM4/papR2 | This work  This work | |
| Plasmids | | **Description** | **Source or reference** | |
| pGM190 | | *Streptomyces-E. coli* shuttle vector*, tsr, aphII*, pSG5 derivative*, tipA* promoter shuttle vector | (Wohlleben, Stegmann, and Süssmuth 2009) | |
| pGM190/papR2 | | pGM190 derivative, PtipA, *tsr, aphII, hispapR2* | (Mast et al. 2015) | |
| pRM4 | | pSET152, *ermEp** derivate (Φ C31 integration vector with mutated erythromycin promoter, *acc(3)IV* with artificial RBS | (Musiol et al. 2011) | |
| pRM4/papR2 | | pRM4 derivative, Φ C31, *acc(3)IV*, artificial RBS, *hispapR2* | This work | |
| Primer | **Primer sequence (5’→3’)** | | | **T_m_ (°C)** |
| For bandshift assays | | | | |
| PredPfw  PredPrv  PredQfw  PredQrv  PpliAfw  PpliArv  Cy5 | AGCCAGTGGCGATAAGGTGTGTGCTCCTCGCGTGAT  AGCCAGTGGCGATAAGGGGGCGGGACGCCCGCCGGG  AGCCAGTGGCGATAAGCGCGGCCCCGCCCGGCGGGC  AGCCAGTGGCGATAAGTTCGCCCAACGCCGCCCCGG  agccagtggcgataagacgggcaaccgtcagttgag  agccagtggcgataagaagtggcccaggttggtcag  AGCCAGTGGCGATAAG | | | 67  81  84  77  76  76  60 |
| For transcriptional analysis | | | | |
| 16Sfw  16Srv  redPintfw  redPintrv  redQintfw  redQintrv  cl3fw  cl3rv  cl6fw  cl6rv  cl9fw  cl9rv  cl10fw  cl10rv  cl15fw  cl15rv | cggccttcgggttgtaaacctc  GCCCCCGTCAATTCCTTTGAGTTT  ggttgaggatgcgggagaag  agatcgacgcggtcatcgtg  cgacaagctcgttgatcttg  gaactcggactggatgac  ttcctgccggacttcatgg  gtgccggttgatcctgttg  gagcgtgagcatcacgttgg  tcgccgaggacaaggacttc  aacctcgcggacctcaccac  ccggtcatgcagagtctcag  ggtgatgccgtacatgttgacc  actcgtacgccttcgacttctc  tcggaggtgctgttcttg  cgaacgcatccacatctg | | | 63  62  58  60  54  52  58  57  59  59  58  60  59  58  55  54 |
| For qPCR analysis | | | | |
| HrdBfw  HrdBrv  redPqPCRfw  redPqPCRrv  redQqPCRfw  redQqPRCrv | CCCTTGGTGTAGTCGAAC  CTGGAGATCATCGCCGAGG  GAGATCGACGCGGTCATC  gatGACCAGGACCCGTTC  CACGCCGAACTCGGACTG  CCAGGAAAGGcggaccac | | | 53  58  56  55  59  59 |


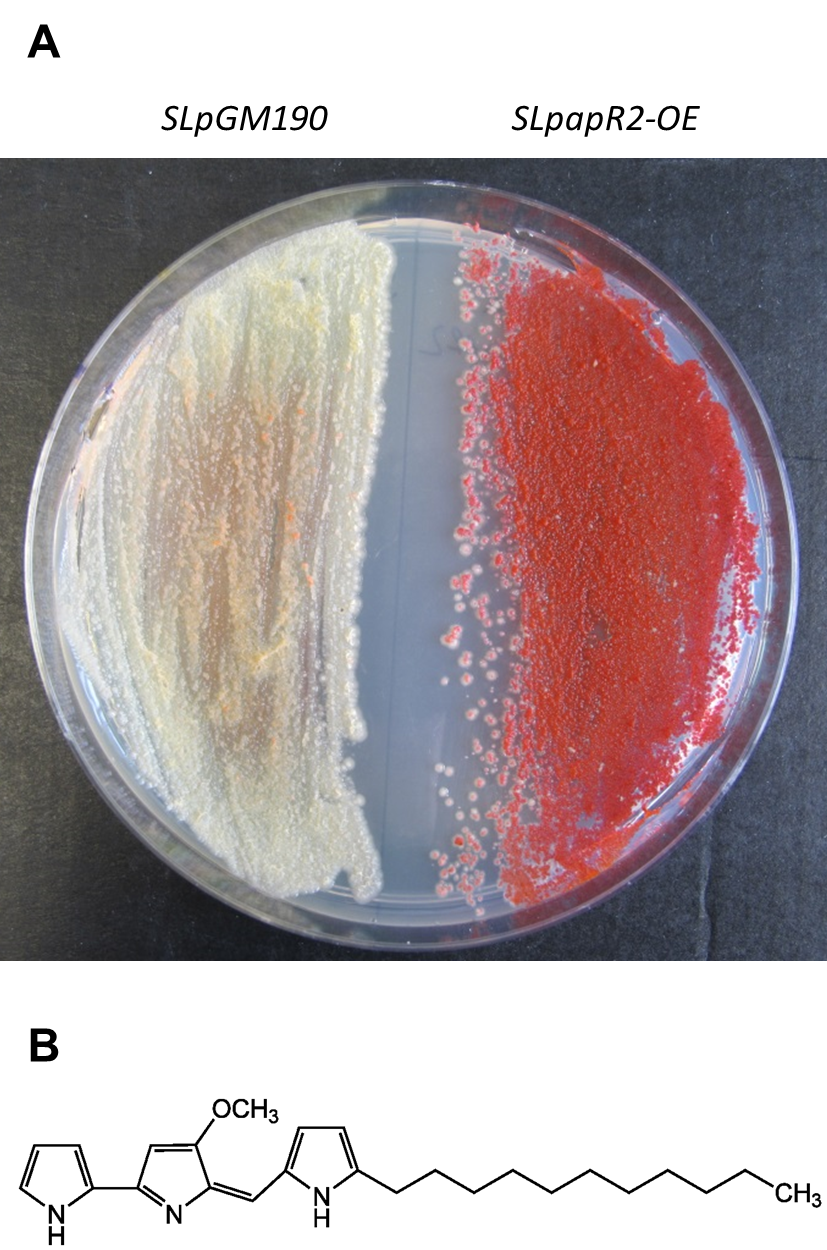


Supplementary Figure S1: Morphological phenotype of SLpGM190 (left) and SLpapR2-OE (right) on R5 agar with thiostrepton 12.5 µg/ml after four days.

**Supplementary Figure S2:** qPCR analysis of samples of *SLpGM190* and *SLpapR2*-*OE*. Graph shows expression levels in a logarithmic scale of the genes *redP* and *redQ* in qPCR reactions with cDNA derived from 72 h samples of cultures of *SLpapR2-OE* taking the expression levels of control (*SLpGM190*) as unity (=1) (n=6).

**Supplementary Table S2:** SARP consensus sequences of SHP 22-7 and their identity to the PapR2 consensus sequence.


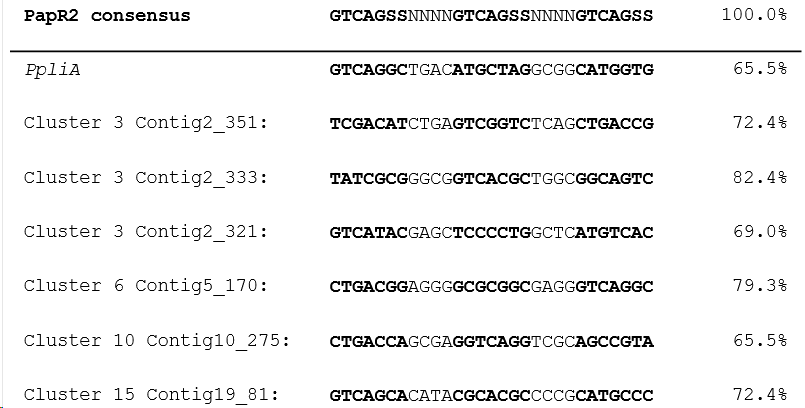


**(A)**


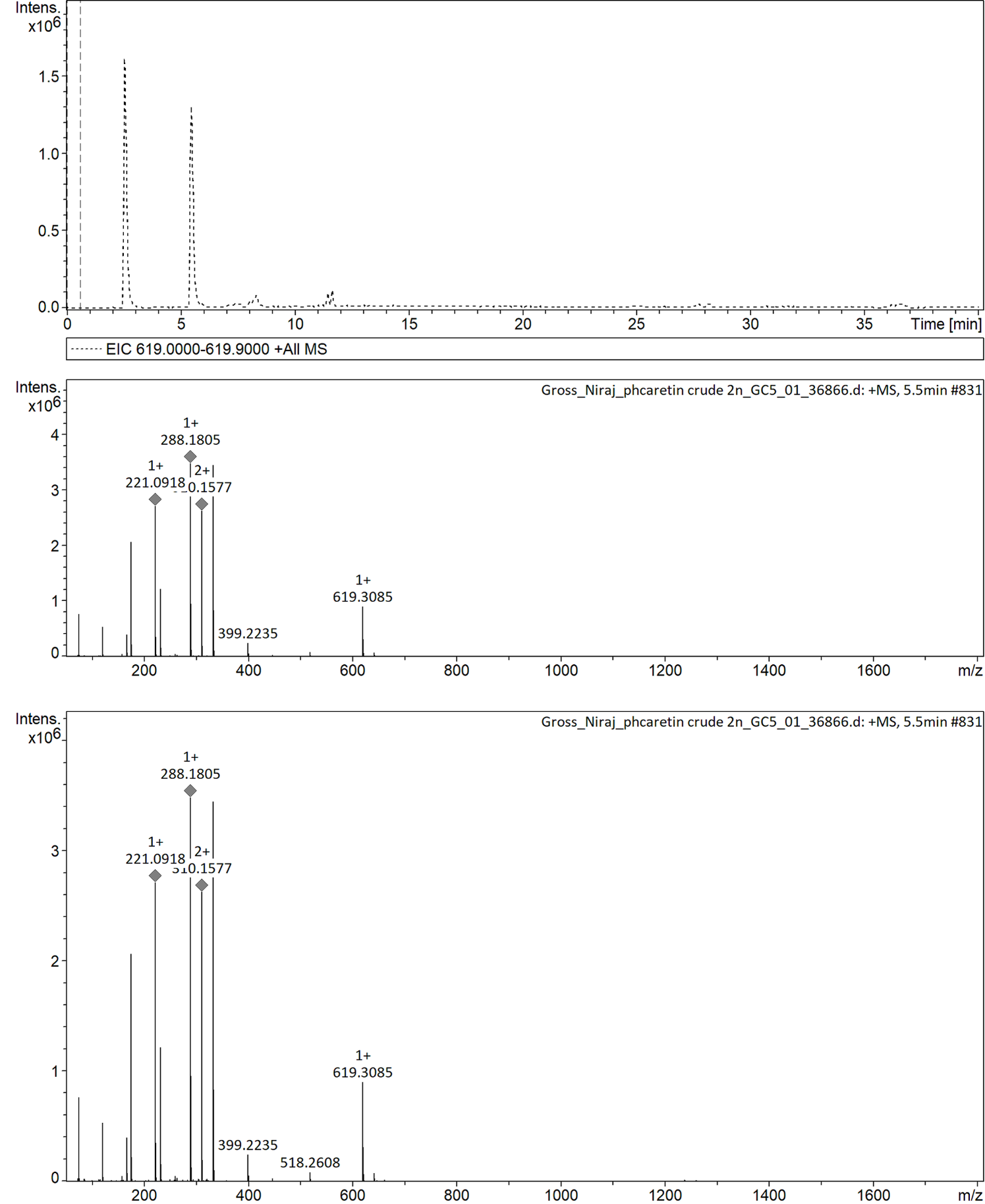


**(B)**

**
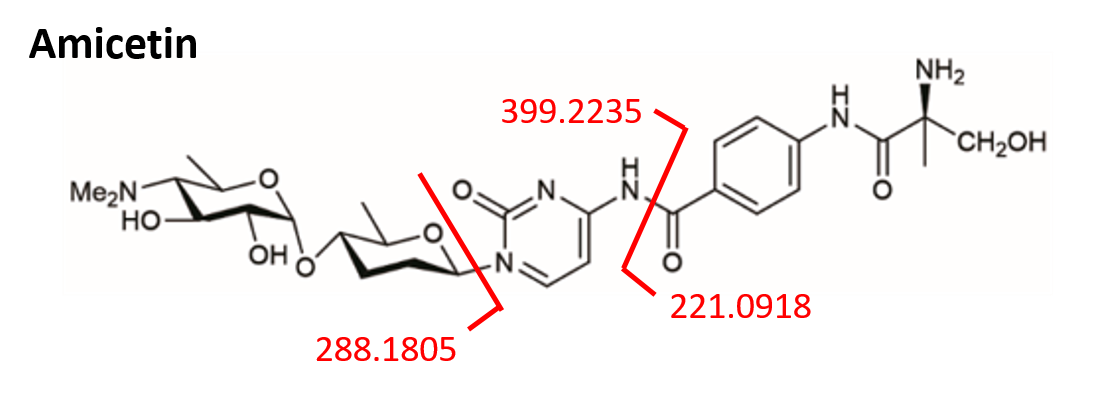
**

**Supplementary Figure S3:** High-resolution MS (HRMS) data for amicetin from SHP 22-7 WT cultured in NL300 (A). Chemical structure of amicetin and expected corresponding fragmentation pattern (b).

.

**(A)**

**(B)**

**
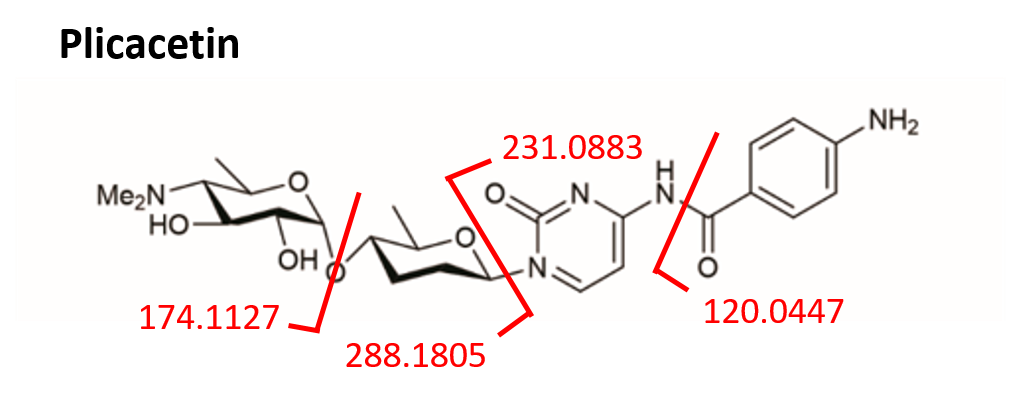
**

**Supplementary Figure S4:** High-resolution MS (HRMS) data for plicacetin from SHP 22-7 WT cultured in NL300 (A). Chemical structure of plicacetin and expected corresponding fragmentation pattern (B).
